# Oral and Enteral Resuscitation of Burn Shock The Historical Record and Implications for Mass Casualty Care

**Published:** 2010-09-01

**Authors:** George C. Kramer, Michael W. Michell, Hermes Oliveira, Tim La H. Brown, David Herndon, R. David Baker, Michael Muller

**Affiliations:** University of Texas Medical Branch (UTMB) and Shriners Burns Hospital, Galveston, and Middlemore Hospital, Auckland, New Zealand

## Abstract

In the aftermath of a mass disaster, standard care methods for treatment of burn injury will often not be available for all victims. A method of fluid resuscitation for burns that has largely been forgotten by contemporary burn experts is enteral resuscitation. We identified 12 studies with over 700 patients treated with enteral resuscitation, defined as drinking or gastric infusion of salt solutions, from the literature. These studies suggest that enteral resuscitation can be an effective treatment for burn shock under conditions in which the standard IV therapy is unavailable or delayed, such as in mass disasters and combat casualties. Enteral resuscitation of burn shock was effective in patients with moderate (10–40% TBSA) and in some patients with more severe injuries. The data suggests that some hypovolemic burn and trauma patients can be treated exclusively with enteral resuscitation, and others might benefit from enteral resuscitation as an initial alternative and a supplement to IV therapy. A complication of enteral resuscitation was vomiting, which occurred less in children and much less when therapy was initiated within the first postburn hour. Enteral resuscitation is contra-indicated when the patient is in “peripheral circulatory collapse”. The optimal enteral solution and regimen has not yet been defined, nor has its efficacy been tested against modern IV resuscitation. The oldest studies used glucose-free solutions of buffered isotonic and hypotonic saline. Studies that are more recent show benefit of adding glucose to electrolyte solutions similar to those used in the treatment of cholera. If IV therapy for mass casualty care is delayed due to logistical constraints, enteral resuscitation should be considered.

## INTRODUCTION

In 1950, the United States first faced the possibility of having to deal with mass civilian casualties on its home soil. US military forces were preparing to lead a United Nations force in combat against a large well-trained army as communist forces were pushing to take over Korea. Russia and China were rapidly developing nuclear weapons with advanced missile delivery systems. In 1950, the U.S. was training soldiers on how to fight on the atomic battlefield, while school children were soon to begin practicing “drop drills” as a proposed means of protection against atomic explosions. In response to these grave events, the Surgery Study Section of the National Institutes of Health published a strong recommendation for a specific treatment of burns and traumatic injuries of mass casualties in a 1950 issue of JAMA.^1^ The following box is the summary statement from this article and is followed by an abstract of the background that led to the summary statement.

**“The use of oral saline solution is adopted as standard procedure in the treatment of shock due to burns and other serious injuries in the event of large-scale civilian catastrophe.” NIH Surgery Study Section of 1950.**^1^

It was the “consensus of the 1950 Surgery Study Section that on the basis of the animal work done by Dr. Rosenthal of the National Institutes of Health and the clinical work done by Drs. Carl A. Moyer, Charles Fox and others^2^^-^9 the efficacy of such treatment had been definitely demonstrated and that, while there is need to stimulate additional research in this field, our present knowledge is sound enough so that action can be taken on this basis.” Specifics of the treatment are the use of a hypotonic (160 mOsm), hyponatremic (Na = 85 mEq) buffered (citrated or bicarbonated) solution, referred to as Meyer's solution, that could be administered by drinking or gastric infusion. Meyer's solution was used in at least three clinical trials.^5^,^10^,^11^ The 1950 Surgery Study Section ended their recommendation “We feel strongly that it is important for the medical profession of the country and for those planning for the handling of potential disasters to be informed of the value of this simple and easily carried out form of treatment.”

For the most part this recommendation and the scientific background supporting oral resuscitation has been forgotten. America did not have to deal with nuclear attack or a mass casualty situation. Further, the development, commercialization, and acceptance of simple-to-place plastic IV catheters along with the rise of new specialties of emergency medicine and trauma surgery led to a focus of providing highly focused and advanced care for the limited number of trauma or burn patients. Mass burn casualties can result from accidents, war, and acts of terrorism. Such threats demand a reconsideration of enteral resuscitation for situations where the number of patients exceeds the resources to provide standard of care.

### The Importance of Early Resuscitation and the Need for Alternatives to IV Therapy

Fluid resuscitation must be started promptly in order to achieve the best outcomes. The high survival rates of modern burn care are attributed to application of prompt and aggressive resuscitation using intravenous infusion of lactated Ringer's.^12^ Half or more of the fluid requirements in the first 24 hrs must be administered in the first 8 hrs for optimal outcomes.^13^,^14^ Studies have shown that severely burn-injured children have increased mortality rates and higher rates of organ failure when initiation of fluid therapy is delayed by even a few hours.^12^,^15^

There is a need to have a better means for the initial resuscitation of burn shock. Most of the world's population does not have access to prompt advanced medical care and IV therapy in the event of severe injury. Burn resuscitation can be delayed in rural regions, in underdeveloped countries, and in a variety of circumstances that delay notification and dispatch or limit medical resources. Initiation of IV access can be particularly delayed in children because of difficulties with cannulating small vessels through burned tissue. At the Galveston Shriners Hospital, referrals of burn-injured children often have a record of delayed resuscitation.

Mass disaster in which the large number of casualties simply overwhelm care providers is another situation where intravenous fluid therapy for the treatment of burn injury will necessarily be delayed. Perhaps at no time in our country's history have we been more aware of the potential for mass casualties; we must plan for the best means to treat thousands of victims of explosions and fires. The September 11, 2001 terrorist attacks caused explosions and fires that resulted in scores of trauma and burn-injury casualties that were effectively treated at New York and Washington trauma and burn centers. However, the medical challenge could have been far greater. If the attacks had come a few hours later or if the World Trade Center towers had not collapsed, there could have been thousands of casualties with large and severe burns requiring prompt fluid resuscitation. Doctors at New York and Washington burn centers contemplated the impossibility of providing prompt resuscitation when dealing with such a number of patients.^16^

Fluid therapy is also a critical component of combat casualty care. Acute hemorrhage is the major cause of death in conventional warfare, accounting for about 50% of all battlefield fatalities as reviewed and analyzed by Bellamy.^17^ Bellamy also noted that administration of effective first aid and resuscitation solutions in the field could reduce battlefield mortality by ∼20%. Unfortunately, fluid therapy for combat casualty care can be delayed, limited, and inadequate owing to logistical constraints for field personnel and supplies and delays in casualty evacuations due to tactical situations.

## THE HISTORIC RECORD OF ENTERAL RESUSCITATION

We have identified multiple reports in the preMedline literature prior to 1966, and in non-English speaking literature that describe enteral resuscitation of burn injured patients. We define enteral resuscitation as drinking in conscious patients who tolerate it with limited vomiting and by gastric infusions in those that did not. Enteral resuscitation can be applied by laypersons without specialized medical supplies of sterile lactated Ringer's or vascular catheters.

Enteral resuscitation has been evaluated in animal experiments^2^,^3^,^18^,^19^ as well as in reasonably large scale trials of burn injured children and adults.^5^,^6^,^10^,^11^,^20^^-^26 These studies appear to have established effectiveness and value of enteral resuscitation using balanced saline solutions. More recent studies on different formulas for the treatment of cholera suggest that solutions containing glucose, amino acids, and starches might have enhanced effectiveness compared to simple electrolyte solutions. The main focus of this review is to critically evaluate the evidence provided by these studies for the effectiveness of enteral resuscitation as well as data on its limitations and dangers.

### Preclinical Studies of Enteral Resuscitation of Burns and Trauma

Sanford Rosenthal was a research pharmacologist at the NIH who conducted an extensive series of experiments dealing with experimental treatment of burns and trauma.^2^,^3^,^19^ Most of the research was done using mouse models of hemorrhage, burn injury, or traumatic shock (tourniquet). Rosenthal's shock model was designed to have a high mortality rate in untreated controls (typically >50% in less than 6-hrs). A consistent finding of these studies was the great survival benefit of large volumes of isotonic NaCl when administered via the oral or intraperitoneal routes. Pure water offered virtually no benefit and 5.5% glucose solutions offered little benefit compared to untreated controls. Effectiveness of plasma or saline administered intravenously was demonstrated, but generally IV infusions were not as effective as that of large volumes of oral saline equal to 100-150 mL/kg for treatment of a 100% TBSA partial-thickness burn and 80 mL/kg for treatment of a 40-50 mL/kg hemorrhage.^2^,^3^

It is surprising that Rosenthal reported that equal volumes of IV saline or plasma were often less effective than enteral administration of saline, but this may reflect that all fluids appear to have been administered as a bolus. An oral (gastric) bolus would enter the circulation over time, while an IV bolus might cause acute hemodynamic overload. We can imagine the cardiovascular effects of a 50-100 ml/kg intravenous bolus that is equal to 1–2 blood volumes. Thus, it is impossible to extrapolate Rosenthal protocols and results against modern standard of care treatments. Nevertheless, the importance of Rosenthal's studies is that they demonstrated an efficacy of gastric infusion and enteral resuscitation in severe shock due to burns, hemorrhage, or trauma, and that they led to several clinical trials.

Plasma was established as the vascular deficit of burn shock in the mid-20th century and this led to the widely accepted belief at the time that intravenous infusion of plasma was the best means to reverse the sequelae of burn shock^31^^-^33 However, evaluations of treating mass casualties at Pearl Harbor and the Coconut Groove fire left burn doctors unsatisfied with the mortality of major burns.^6^ There were few basic science studies at this time, but the studies published on burn resuscitation focused on the value of plasma vs saline. Rosenthal,^3^,^19^ and Baxter and Shires^34^,^35^ were pioneers when they suggested that large volume resuscitation with crystalloids, specifically saline or LR, was more beneficial than plasma for treatment of burns and hemorrhage.

### Clinical Studies Using Oral and Enteral Resuscitation of Severe Burn Injury

Enteral resuscitation of moderate to severe burn injury has been described in twelve studies that we have identified; listed here and in Table 1, Fox (1944), Moyer (1949), Markley (1956), Wilson and Stillman (1960), Davies (1964), Franke (1964), S0rensen (1968), Jackson (1966), Monafo (1970), Ahnefeld (1975), Maksimov (1989) and El-Sonbaty (1991).^5^,^6^,^10^,^11^,^20^^-^26,36 Despite this background few surgeons or intensivists consider oral or enteral resuscitation as even partially supportive for treatment of severe hypovolemia. Results are summarized in Table 1 and discussed to include both benefits and complications of each clinical study of enteral resuscitation of burn injury that we have found. The patient numbers (n=) refer only to patients treated with enteral resuscitation and do not include numbers from control groups. We found no published clinical studies on enteral resuscitation of trauma or hemorrhagic hypovolemia.

In 1926 Davidson reported how burn patients in his day often had hyponatremia and hypochloremia.^37^ Subsequently, he reported efficacy with large, as much as 10 liters, infusions of isotonic saline administered in first 24-hrs by three routes, subcutaneous, intravenous and rectal.

The first published study we identify describing the exclusive use of oral or enteral resuscitation of severe burns was by Charles Fox in 1941. Fox used oral resuscitation treatment exclusively for both partial and full thickness injuries, but he reported details on nine severely injured patients with moderate to large full thickness injuries. Four children (TBSA 23%–80%) and five adults (19%–41%) with full thickness burns and who exhibited signs of shock were treated by Fox with chilled isotonic sodium lactate given upon admission and thereafter at 15 minute intervals to administer a total 100-150 mL/kg in 24-hrs.^6^^-^8 As calculated per the Parkland formula, this volume would be considered sufficient by current standards to resuscitate a 25–38% TBSA injury. Vomiting, which occurred frequently in severe burns, was treated by administration of more fluid and in these patients nasogastric tubes were used to provide continuous fluid delivery. Fluid infusions were adjusted to obtain 1–2 liters of urine per day. When food was tolerated, a high protein diet was started.

Fox reported “In general the large volumes of sodium lactate were well tolerated, the patients wanted water to drink, but after a short time became accustomed to the lactate and drank copiously of their own volition.”

In 1949, Moyer reported on his approach to oral resuscitation used to treat more than 30 severely burned children and adults.^5^ Moyer found that NaCl alone caused an acidosis, which could be prevented with bicarbonate, lactate, or citrate. These buffered and balanced saline solutions were also more palatable and induced fewer indices of nausea and vomiting. Moyer suggested that the citrated formula was perhaps the most palatable. Moyer was careful to state that when circulatory collapse was present, it was necessary to administer IV fluid with lactated Ringer's or plasma until the peripheral collapse was corrected after which oral resuscitation was initiated. Moyer did not present any quantitative guidelines to determine at what level of shock enteral resuscitation is contraindicated and at what level it becomes effective.

In 1956, Markley et al. reported on the results from an extensive NIH sponsored trial conducted in Peru that evaluated the use of oral resuscitation. This study was one of the only controlled trials of oral resuscitation in which half the patients received IV plasma and blood per the standard of care of his day. Treatment was alternated for successive patients between oral saline or IV therapy using plasma and saline. Markley used an isotonic bicarbonated saline administered solely or chiefly orally as the emergency treatment in the first 48-hr for severe burns. Entry criteria were TBSA > 10% with mean burn size being ∼40% and ∼30% in 55 children and 56 adults, respectively. Control groups of comparable size were administered IV plasma or blood, and some saline with volume determined per the Evans formula. By modern standards we would consider the 1-ml/kg volume of the Evans formula to be inadequate volume.

The volume of oral saline was 110 mL/kg in the first 24-hrs. The 48-hr mortality data were equal or better with oral resuscitation, while data on hematocrit and urine output suggested effective volume expansion and improved hemodynamics with oral resuscitation.

Markley's mortality data with oral resuscitation was similar to standard of care mortality results reported from U.S. urban burn centers of the day. However, urine output and mortality data grouped for different size burns suggest an inability to fully resuscitate patients with burns >50% TBSA. The dose of fluid administered was independent of burn size, 110 mL/kg during the first 24-hr. This is a volume equivalent to the Parkland formula for a 27% TBSA injury. Thus, we could expect that inadequate volumes may have been administered to Markley patients with burns larger than 30% TBSA. Two unanswered questions are: would outcomes be improved with oral administration of larger volumes, and what is the limit on volume that can be delivered to a severely burn injured patient via the enteral route?

In 1960, Wilson and Stirman reported on outcomes of 142 burn-injured patients with 15-60% TBSA injuries treated with isotonic saline compared to saline plus blood.^21^ Of course, blood was administered via IV route. Mortality rate was higher in the group that received blood and Wilson made the case that blood was rarely needed for burn resuscitation. All patients were treated with oral intake of the salt solution with IV being used initially only if the patients demonstrated peripheral circulatory collapse, defined as low or no blood pressure. When oral treatment seemed inadequate to maintain the circulation or impractical because of vomiting, these patients were administered IV lactated Ringer's solution or plasma until stabilized. However, 81 of Wilson's patients were treated exclusively with oral intake including some patients with 60% TBSA injury. It is unclear if Wilson's patients may have also received some nutrition that would have provided glucose or other absorbable solutes. Most subsequent clinical trials of enteral resuscitation reported on the use of oral delivery of bicarbonated, citrated, or lactated saline solutions that were more palatable, and associated with less nausea and vomiting.

Davis reported in 1964 for the British Medical Research Council on the increased use of IV plasma for burns that was made available by Britain's National Blood Transfusion Service (NETS). The NETS was launched during the London Blitz in World War II, and to this day provides British hospitals with an adequate supply of low-cost blood products. The focus of the Davis study was on isotope measurements of RBC volume and plasma volume in burn patients, 10-95% TBSA, subjected to routine care which included oral water and oral Moyer solution administered at 2x normal oral fluid input, a rate independent of injury size. Patients with burns of 40% TBSA or less were resuscitated initially with oral crystalloids. This volume was clearly inadequate in several patients as half of these required subsequent IV saline or saline plus plasma, started when vomiting was excessive or clinical condition deteriorated. On the other hand, in half of the patients oral resuscitation was adequate with good clinical outcomes.^10^

Franke and Kock-Marburn reported in 1964 on the first clinical use of oral electrolyte solution that also contained glucose for treatment of severe burn injury in children in the German language journal, Langenbeck Arch Klin Chir.^22^ This group often exclusively used gastric infusion and an anti-emetic to treat 19 out of 22 children with 8 to 38% TBSA injuries, all of whom survived. One 2 year old with a 31% TBSA burn including 7% full thickness received 3,000 mL and 2,300 mL enterally on day 1 and day 2, respectively. Three additional children with larger burns of 35 to 70% received IV fluid and gastric infusions and two of these died from cerebral edema and heart failure.

In 1966, Jackson and Chen^11^ set out to determine if a moderate sized burn could be safely treated enterally with Meyer's solution, since most burn centers advocated IV plasma. Out of 162 burn cases (113 children and 49 adults) with 10% to 35% TBSA injuries, 75% were exclusively treated with oral resuscitation, 17% received IV LR infusions and 8% “required” IV colloid infusions. Vomiting occurred in 30% of the children and 51% of the adults and thus vomiting was not necessarily the criterion to stop oral resuscitation or start IV therapy. Mean urine output was reported less than normal in 73% of the patients at 8-hrs postburn, with this number dropping to 44% at 24-hours and 34% at 448-hours. Children had a substantially lower percentage of oliguria than in adults. This study suggests better toleration and efficacy of enteral resuscitation in children compared with adults. Similar conclusions were recently reached by Brown et al. who showed younger children have less vomiting with enteral feedings.^38^

Based on the published levels of urine output in most pre-1980 studies, it is apparent that severely injured (>50% TBSA) burn patients were often under-resuscitated compared to our current standards. Burn patients almost always requested water to drink and while not a stated part of clinical care, drinking water was allowed in early burn care. This was often a mistake as significant water consumption without sodium supplementation was shown to cause an early “toxemic phase of burn injury” due to water intoxication.^5^ Paracelsus (1493-1541) noted that severe burn injury was accompanied by fever and a ‘drying out of the blood’, resulting in thirst. However, he recommended that if a patient drinks, death will follow. Drinking Na+ free fluids can cause severe hyponatremia and cerebral edema. On the other hand, drinking electrolyte solution is an effective means of burn resuscitation. Animal and clinical studies both showed the critical value of replacing oral water with oral salt solutions.^2^,^5^,^6^,^20^

A novel approach to oral resuscitation that might allow safe drinking of non-sodium-containing beverages was suggested by Sørensen who sought a means to treat mass casualties due to thermal nuclear warfare. The impetus for his work was the U.S.-Cuban missile crisis of 1962. Subsequently, Sørensen resuscitated 26 consecutive patients by allowing them to drink any beverage they wanted, which reportedly was tea, beer, milk, or water. Sørensen reported that salt solutions had a disagreeable taste and thus administered 7.5-g salt tablets 5-g NaCl and 2.5-g NaHCO3 per each liter of fluid. Similar salt tablets have been commercially available, but currently are difficult to find. Sørensen setup an IV drip if needed, but most of his patients did not require it. Children and adults could take the tablets. Fluid intake was not controlled per burn size and totaled 150 to 200 mL/kg in first 24-hrs and 100 to 150 mL/kg in the second 24-hrs. Eighty percent of these patients required no IV support and this group had burns as large as 45% TBSA. To our knowledge, this unique approach to mass casualty care was never followed up or used again.

In 1970, Monafo reported on resuscitation after major burn injuries using 600 mOsm hypertonic lactated saline (HLS) in which patients were orally and intravenously resuscitated.^24^ Monafo used HLS to partially resuscitate 4 burn adults with 30% to 95% TBSA burns and 3 children with 22% to 58% TBSA burns. Most were full thickness injuries. Patients received 10-60% of their fluid needs in the first 24 hrs from oral fluids and 60-90% of their fluid needs in the second 24 hrs. This report shows that partial oral resuscitation of severe burns can be successful and that it can be accomplished with a hypertonic solution without glucose. In subsequent years Monafo continued to use IV hypertonic saline, but his use of oral resuscitation was not continued. To our knowledge, Monafo's suggestion for efficacy of oral hypertonic saline has never been followed-up either clinically or experimentally.

Rosenthal had found hypertonic saline to be less effective than isotonic saline treatment of burn-injured mice. Other early studies reported that hypertonic saline was less palatable and led to greater incidences of vomiting. However, Monafo stated, “it was discovered that HLS solution (iced to be made palatable) was easily tolerated orally in significant amounts without apparent GI dysfunction.” One wonders if the ice not only chilled, but also significantly diluted the concentration of the isotonic saline. Hypertonic fluid that enters the intestine rapidly becomes isotonic due to mobilized fluid, but thereafter can be rapidly absorbed along with its sodium, particularly if mucosal blood flow is enhanced as has been reported after hypertonic resuscitation using IV infusion.^39^

In 1975, Ahnefeld reported on the use of Liquidsorb, a slightly hypertonic 370 mOsm electrolyte ‘lemonade’, for treatment of 68 burn patients with 12 to 34% TBSA injuries.^36^ Liquidsorb contained 4 g of glucose, 100 mg ascorbic acid, and 12 mg nicotinamide per liter of bicarbonated saline. Only 4 of 58 who arrived at the hospital within one hour of injury could not drink the solution. However, 16 of 23 patients who arrived more than 2-hours after injury were not able to drink because of “sickness, vomiting, centralization of the circulation, and unconsciousness.” These data suggest that in order to be effective, oral resuscitation must be started early, ideally within the first hour after injury.

Ten subsequent burn patients with 12 to 26% injuries were also treated with Liquidsorb and demonstrated good absorption of one liter in the first hour and 750-mL in the second hour when delivered by stomach tube in 50-mL increments. This approach was well tolerated except for patients that had “centralization” or peripheral vascular failure as evident by collapsed peripheral veins and delayed capillary refill. However, oral resuscitation was effectively implemented in patients that were initially resuscitated with IV therapy and after reestablishment of “normal circulatory conditions”.

### Modern Studies of Enteral Resuscitation of Burn Shock

Surgical textbooks and the Advance Burn Life Support (ABLS) course suggest that oral fluids are an adequate means to treat volume losses in small burns of 5-10% TBSA. Recently, Brown et al have suggested considering enteral resuscitation on larger burns up to 40% TBSA alone or used in conjunction with IV fluids.^38^ We suggest that TBSA should not be the sole criterion for the appropriateness of enteral resuscitation. Larger but less severe burns should do well with enteral resuscitation. While smaller burns with deep injury, particularly with additional trauma, could require IV fluid therapy. There is no consensus on how best to perform enteral fluid therapy with small burns. A recent survey of burn centers in the United Kingdom shows a wide divergence of fluids used for oral resuscitation of 5% to 10% TBSA burns including electrolyte solutions and beverages (i.e. water, milk and juice).^40^

We could identify only a few publications in the last 20 years on enteral resuscitation of large burns and none were in Medline referenced literature. El-Sonbaty reported good results with oral resuscitation of 20 children with 10-20% TBSA burns using the WHO ORS.^26^ The depth of the burns was not presented, but we can assume that most of the patients did not present in burn shock as the children's mothers under the supervision of the nurse administered the oral hydration. The volume and rate of oral hydration with WHO ORS used by El-Sonbaty was identical to standard Parkland formula for IV administration. He reported oral hydration to be as effective as IV lactated Ringer's, also administered at Parkland rate. Again there is no way to accurately compare relative vascular expansion or effectiveness on the two groups. Burn injuries of this small size do not induce hypovolemic shock. Patients in both groups developed hyponatremia (125-130 mEq/1) on day 5 post-injury, but otherwise had unremarkable outcomes. A comparison of compositions of oral hydration solutions versus IV solutions shown in Table 2 suggests that hyponatremia may be a problem with most oral solutions and that oral resuscitation of burned patients may require a solution with a higher sodium concentration, such as used by Monafo.

Jiang reported the only animal study of oral resuscitation of severe burn injury that we have found in which the control group was treated with the modern Parkland formula.^18^ Anesthetized dogs were inflicted with a 30% partial thickness burn and then treated with a 347 mOsm “burn drink” of glucose NaCl and NaHCO3 (Table 2). Total volume administered over 24 hrs was the Parkland formula, 4 ml/Kg per % TBSA. Controls were untreated burns, and burns treated with a 1/10 dilution of the burn drink (35 mOsm). Impressive improvements in plasma volume, cardiac output and urine output were shown, but only for the 347 mOsm “burn drink” group, not for the hypotonic enteral group.

Despite a fairly extensive literature on enteral resuscitation of burn shock, most present-day clinicians are unaware of its utility as an option, except in minor burns where resuscitation is not critical. However, two modern clinical trends speak to the question of efficacy and safety of enteral resuscitation. These are oral hydration in the treatment of cholera, and aggressive enteral feeding of trauma patients.

### Treatment of cholera

Oral hydration has been a focus of basic and clinical research for the treatment of life threatening dehydration due to diarrhea. Dehydration and associated shock due to cholera and diarrhea are the leading causes of pediatric mortality in the world.^41^ There have been dramatic reductions in the mortality rate of cholera victims in underdeveloped countries due to oral rehydration solutions that are easily administered by the patient's family or other lay persons. The successful use of glucose-salt solutions such as the WHO ORS has been called one of the major medical advances of the 20th century based on the tens of millions of lives saved.^41^ Hydration with pure water is an ineffective treatment of cholera, but providing substrate for the intestine's coupled sodium-glucose transporter provides a means to replace electrolytes, energy substrate, and water in severe dehydration. Specifically, WHO ORS packets provided by the World Health Organization and governments have provided a low cost and highly effective treatment of dehydration due to cholera. Children and their mothers in under-developed countries are very familiar with the WHO ORS packets and how to make up the solutions and administer them to treat dehydration due to diarrhea. Most trained specialists in the U.S. are unfamiliar with WHO ORS and treat dehydration exclusively with IV infusions.

### Early enteral feeding of burn injury

Recent research has established significant clinical advantages of early intestinal or enteral resuscitation even if it can only be performed in hospitals. Enteral feeding may protect the gut by enhancing or maintaining the mucosal barrier, which can be a route for systemic entry of bacteria and endotoxins. While early enteral feeding has been traditionally avoided in burn patients, it has recently been demonstrated to be safe and effective for nutrition when started immediately with hospital care of patients with large burns^42^ or used before, during, and immediately after surgery of burned patients.^18^ The gut benefits from early enteral resuscitation, particularly when specific nutrients are included. Enteral resuscitation increases intestinal blood flow and results in better maintenance of gut barrier integrity. This may reduce the risk of sepsis and multi-organ failure.^43^

### Composition of Oral Hydration Solutions

The optimal composition of oral replacement fluid has not been determined and will likely vary for different indications. The World Health Organization has had great success with slightly hypertonic ORS solution containing glucose, sodium, chloride, and bicarbonate. It is easily formulated by adding WHO ORS powder packets to water, Table 2. Gatorade®, PowerAde® and other “sports drinks” are low in sodium (5-20 mmol/L), as they are designed to replace perspiration losses, as well as to increase palatability.^44^ Monafo (1970) utilized a 600 mOsm hypertonic lactated saline solution (HLSS) containing sodium, racemic D-L lactate and chloride to orally resuscitate 10 burn injury patients.^24^ It seems logical that a solution similar to Ringer's lactate or HLS with the addition of glucose with an osmolarity range of 260-330 mOsm/L given by drinking or infusion through a nasogastric tube could rapidly be absorbed and provide the large volume and Na necessary for burn resuscitation. Although such a formulation appears logical, it will require substantial research to define an optimal solution.

### Sports Drinks

Oral hydration in developed countries has not focused on treatment of disease, but rather on sports drinks, such as Gatorade, to provide effective hydration of people engaged in strenuous activities. The financial success of the sports drink industry with sales of 1.5 billion dollars per year has stimulated both research and the creation of numerous marketed drinks for hydration and nutrition. The hydration effect of such drinks is based on the same principles as those of the WHO ORS.^45^,^46^ The higher sodium concentration of WHO ORS versus Gatorade reflects the different electrolyte losses associated with diarrhea and sweating, respectively. Sweat has a sodium concentration of 30-60 mEq/L, which is less than the 60-120 mEq/L in watery stools or of the 130 mEq/L of plasma extravasated into a burn wound. Resuscitation of severe hypovolemia as with burn shock is a greater challenge than dehydration due to sweating or diarrhea because effective solutions used for volume expansion, such as LR, require a higher sodium concentration than most modern oral hydration formulas and could require delivery of larger volumes. On the other hand, Table 1 shows that both isotonic and hypertonic solutions without glucose and with higher sodium concentrations were effective for resuscitation of severe burn shock.

### Oral Hydration to Expand Astronaut's Blood Volume

NASA has employed different oral hydration strategies for astronauts prior to shuttle reentry to expand blood volume and prevent orthostatic hypotension during descent. Oral rehydration with salt water or with water and salt tablets was shown to expand blood volume as a counter measure against the loss of vascular volume and cardiovascular tone that occurs during prolonged space flight. NASA's Greenleaf et al studied formulations with differences in osmolarity and sodium concentrations.^47^ They concluded that cation content is more important than osmotic content for plasma volume expansion. This led to the development of AstroAde a high-sodium oral hydration solution (Table 2). AstroAde was effective at expanding plasma volume of dehydrated subjects during exercise.^48^ Table 2 compares the compositions of selected oral hydration solutions and IV solutions.

## CHALLENGES AND POTENTIAL LIMITATIONS OF ENTERAL RESUSCITATION

Many studies have been conducted on the development of better IV solutions to treat circulatory shock. However, the hypothesis that enteral resuscitation could play a role in initial resuscitation has not yet been tested against modern resuscitative regimens. Oral fluids have traditionally been considered contraindicated before surgical stress and for patients in hemorrhagic shock, burn shock, or trauma. Enteral solutions may never be recommended with abdominal or thoracic trauma. However, in many forms of trauma and burn injury there should be no direct compromise of the GI tract.

Paralytic ileus and reduced gastric emptying may limit enteral resuscitation. Wilson reported gastric dilation during resuscitation in some burn patients when treated with oral 0.9% NaCl, whereas vomiting was reported in several studies.^6^,^11^,^50^

Vomiting is likely to be the most often encountered complication of enteral resuscitation. Fox reported that vomiting was a frequent occurrence in the most severely burn-injured patients treated with an isotonic buffered saline solution. However, incidences of vomiting are greatly reduced when resuscitation is started in the first postburn hour.^36^ Burn doctors at UTMB and Shriners Hospital, Galveston have reached a similar conclusion for enteral feeding. Enteral feeding via gastric or intestinal infusions are started as soon as possible after admission with a high success rate, but when enteral feeding is delayed paralytic ileus is a larger problem. Children appear to tolerate oral resuscitation better than adults as they have a significantly lower rate of vomiting than adults.^38^ Vomiting was also reported to be reduced by using chilled solutions, hypotonic solutions, or by buffering solutions with citrate, lactate or bicarbonate.^5^,^6^,^24^ We suggest that administration of anti-emetics and other pharmacological agents might enhance gastric emptying and gut motility and may further reduce vomiting. The use of antihistamines and antacids are standard treatment to prevent stress ulceration and may help aspiration.

There also may be advantages to early intestinal or enteral resuscitation even if performed only in conjunction with IV infusions. Enteral feeding may protect the gut by enhancing or maintaining the mucosal barrier, which can be a route for systemic entry of bacteria and endotoxins. While early enteral feeding has been traditionally avoided in burn patients, it has recently been demonstrated to be safe and effective for nutrition when started soon after hospital admission in patients with large burns^38^,^42^ or used before, during and immediately after surgery of burned patients.^18^ At UTMB and Shriners Hospitals all burn patients and some trauma patients are started on enteral feeding in the first two hours of admission. It is possible that the gut will benefit from early enteral resuscitation, particularly when specific nutrients are included in the formulation. Oral resuscitation may increase intestinal blood flow and result in better maintenance of gut barrier integrity.

There are serious concerns regarding the early feeding of patients after trauma and surgery. Vomiting, aspiration, and pneumonia can be life-threatening complications. Many trauma patients have full stomachs upon injury; however, data suggest that aspiration in trauma patients is a rare occurrence.^30^,^51^ Early trauma care typically involves high doses of analgesics and procedures including surgery that along with the stress of shock can induce paralytic ileus.^52^,^53^ Stress ulcers due to mucosal ischemia and gastric acid are another complication of burn and traumatic injury. Most oral rehydration solutions (ORS) are buffered alkaline solutions that may protect against the formation or severity of ulcers.^54^

Clearly, the dangers of enteral resuscitation must be weighed against the benefits. However, we suggest that specific solutions and administration regimens including pharmacological agents and perhaps special nasogastric (NG) tubes will permit safe implementation of enteral resuscitation.

Enteral resuscitation is likely to be contraindicated in severe shock when intestinal blood flow is so low as to prevent any significant fluid absorption. Clearly, there is a limit to the effectiveness of enteral resuscitation based on the severity of shock. Several of the clinical trials state that enteral resuscitation was not effective when there is “centralization of the circulation” or “peripheral vascular collapse”.^5^,^21^,^22^,^55^ Ahnefeld defined “peripheral vascular collapse” as a condition in which peripheral veins are collapsed and capillary refill times in nail beds are greatly lengthened.

The disappearance of enteral resuscitation from contemporary medical consciousness is largely due to two factors: 1) The development of plastic IV catheters. 2) The rise of critical care medicine and trauma specialists with a focus on advanced team care of individual patients. Physicians receive limited training or practice on how to deal with mass casualties. The option of enteral resuscitation as a clinical treatment for burns has been bypassed and its utility is largely forgotten. The Korean War was the first military engagement in which rapid helicopter evacuations and resuscitation was implemented along with advanced care performed at mobile army surgical hospitals (MASH). Most certainly, intravenous infusion is a direct solution for correcting intravascular volume deficit. The value of prompt resuscitation by IV infusion greatly decreased incidences of renal failure. The Korean war experience helped define our modern urban system of paramedics, rapid patient transport, and trauma centers. Unfortunately, there always will be scenarios where IV fluid cannot be administered promptly or at all. The events of September 11, 2001 underline the importance of having an effective and logistically feasible means to provide initial fluid resuscitation to mass casualties. For such scenarios we need to reconsider enteral resuscitation and to define its efficacy and limitations.

## CONCLUSIONS AND RECOMMENDATIONS FOR CLINICAL USE

Our recommendation is to use standard of care IV resuscitation for resuscitation of burn shock as advocated in the ABLS course. However, in scenarios of delayed IV fluid therapy when the patient is conscious and needs volume support and has no apparent or suspected GI injury, oral resuscitation is recommended. Further, we suggest oral resuscitation could be used alone in many patients and supplemented or replaced by IV in those patients with the most severe injuries. Enteral resuscitation appears to be much more effective when started within the first hour after injury. Enteral resuscitation may be more effective in children compared with adults. Fluid can be delivered in small sips by drinking or delivered by spoon with a fixed volume of one cup or ∼250-mL to be consumed every 15 minutes. If this is well tolerated, the drinking can be increased to 6 cups/hour. If not tolerated, gastric infusion through an NG tube may be effective. Fox did not consider vomiting a contraindication to enteral resuscitation.^6^,^20^

We know of no definitive studies comparing the effectiveness of the many different formulations used for burn resuscitation. Thus, at present no definitive best enteral solution is identified. However, based on available evidence we suggest solution compositions similar to WHO ORS, which is available at pharmacies and medicine cabinets in most countries, or made-up in the kitchen from the formulas shown in Table 3. WHO ORS is almost always sold as a small sachet of dry ingredients or concentrated syrup to be added to a measured volume of clean water. In the industrialized countries, WHO ORS is not available *per se*, but Rehydrate™ or Pedialyte are similar products available already made up to the concentration to use in 500-mL or 1-liter bottles. Alternatively, solutions can be made in the kitchen from common household sugar, salt, and baking soda added to clean water or Gatorade or lactated Ringer's depending on what is available.

Drinking pure water is NOT recommended, although a limited volume initially would likely do little harm. Large volumes cause hyponatremia and severe complications of water toxicity, hyponatremia and cerebral edema. There are two strategies that may allow the consumption of fairly large amounts of water and related fluids such as tea or colas or juice. Sørensen recommended that a 5 to 7.5 g salt-tablet could be taken with each liter of water for treatment of mass casualties. Treharne suggests that eating a normal diet would be possible in some patients: this would provide the salt needed along with the extra water that would be consumed with the food.^56^

## RECOMMENDATIONS FOR FUTURE RESEARCH

Research in both animals and patients is needed to evaluate the limits of effectiveness, dangers, and complications. The concepts of initial fluid resuscitation and early enteral feeding of burn shock may someday be viewed from a single perspective as two complimentary therapies. However, if burn physicians are called upon to deal with such a mass casualty scenario we suggest that oral resuscitation be considered.

Simply stated, effective resuscitation of small, moderate, and sometimes severe burn injury can be accomplished by either drinking or gastric infusion of buffered saline solution. We suggest that there is sufficient data to justify the application of enteral resuscitation of burn shock in those conditions in which IV fluid cannot be utilized. Further, we suggest that research is needed to define the full utility and limitations of enteral resuscitation.

## Figures and Tables

**Table 1:** Enteral Resuscitation of Burn Injury - the Clinical Record

First author, Date	*n* =	TBSA burn	Solution	Results/comments
*Fox, 1944*	*9*	*19%–80%*	*Isotonic sodium lactate*	*Less vomiting with sodium lactate than other sodium salts. Hypochloremic on day 2*.
*Moyer. 1949*	*30*	*Not listed*	*Hypotonic citrated or bicarbonated NaCl*	*Hypotonic solutions reported to have low incidence of nausea and vomiting. Used IV LR & oral buffered saline*.
*Markley, 1956*	*111*	*>10%*	*Isotonic bicarbonated NaCl*	*Vomiting less in oral group, 55% of cases used oral fluids only, rest received some IV LR or plasma*
*Wilson, 1960*	*142*	*15–65%*	*0.9% NaCl*	*Patients denied oral resuscitation if peripheral vascular collapse, vomiting, or gastric dilation*.
*Davies, 1964*	*20*	*10–40%*	*Mayer's solution bicarbonated NaCl*	*Always started with oral resuscitation, but about half required IV LR due to vomiting or deteriorating shock*.
*Franke, 1964*	*22*	*8–70%*	*Glucose-HCO3 electrolytes*	*Used gastric infusion and anti- emetics. 19 (86%) received only enteral fluids*.
*Sørensen, 1965*	*26*	*10–45%*	*Clear fluids + salt tablets*	*Administered water (100 to 200 mL/kg first 24-hrs) & 7.5-g salt tablet per L*.
*Jackson, 1966*	*162*	*10–35%*	*Mayer's solution Bicarbonated NaCl*	*75% treated with oral fluids only, 25% also received IV plasma or LR. Vomiting occurred in 36% of patients*.
*Monafo, 1970*	*7*	*22–95%*	*Hypertonic lactated saline - 600 mOsm*	*Combined oral & IV treatment with hypertonic lactated saline (HLS)*
*Ahnefeld, 1975*	*68*	*12–34%*	*Slightly hypertonic glucose electrolyte solution*	*Toleration of gastric infusion of 1L was 97% of patients in first hr postburn, but only 30% at 1.5–3 hrs postburn*.
*Maksimov, 1989*	*92*	*10–50%*	*Isotonic bicarbonated NaCl*	*Used enteral resuscitation exclusively in 12 patients w/moderate burns & to supplement IV therapy in 80 patients*
*El-Sonbaty, 1991*	*20*	*10–20%*	*WHO ORS*	*Control group w/ LR - Parkland formula had equivalent outcomes*
*Total*	*709*			

**Table 2:** Oral Hydration Solutions Compared to IV Solutions

Beverage	Carbohydrate	Na+	Cl-	K+	Buffer	Osmolality (mOsM)	Use
	*mM (% wt/vol)*						
*WHO ORS*	*111 (2.0)*	*90*	*80*	*20*	*30*	*331*	*Cholera*
*Gatorade*®	*∼250 (4.5)*	*20*	*20*	*3*	*3*	*280*	*Sports*
*Pedialyte*®	*139 (2.5)*	*45*	*35*	*20*	*30*	*250*	*Dehydration*
*Rehydralyte*®	*139 (2.5)*	*75*	*65*	*20*	*30*	*325*	*Dehydration*
*Fox 's Na Lactate*	*0*	*161*	*0*	*0*	*161*	*321*	*Burn*
*Mayer's CitratedNaCl*	*0*	*85*	*63*	*0*	*29*	*160*	*Burn*
*Monafo 's HLS*	*0*	*300*	*200*	*0*	*100*	*600*	*Burn shock*
*Liquidsorb*®	*222 (4.4)*	*60*	*44*	*4*	*28*	*-370*	*Burn*
*Jiang 's Burn Drink*	*252 (5.0)*	*48*	*28*	*0*	*20*	*347*	*Burn dehydration*
*Ricelyte*	*(3.0)*	*50*	*45*	*25*	*34*	*200*	*Dehydration*
*AstroAde (NASA)*	*0*	*164*	*76*	*0*	*40*	*253*	*PV expansion*
*IV Solutions*							
*Lactated Ringer's*	*0*	*130*	*109*	*4*	*28*	*270*	*PV expansion*
*0.9% NaCl*	*0*	*154*	*154*	*0*	*0*	*308*	*PV expansion*
*Plasmalyte-R*	*0*	*140*	*98*	*5*	*50*	*294*	*PV expansion*

**Table 3:** Oral Resuscitation Formulas–Kitchen or Homemade WHO ORS, Sodium enriched Gatorade and sugar enriched LR

Base ingredient	Volume	Sugar	Salt	Baking Soda
*Clean water*	*1 liter*	*8 tsp*	*1/2 tsp*	*1/2 tsp*
*Clean water*	*1 quart*	*9 tsp*	*2/3 tsp*	*2/3 tsp*
*Gatorade*	*Quart bottle*	*no addition*	*Add 1/4 tsp*	*Add 1/4 tsp*
*Lactated Ringers*	*1 liter*	*8 tsp sugar or glucose*	*no addition*	*no addition*
*Note: Non- sterile* — *Enter al use only not for IV use!*				
